# Liraglutide improves metabolic parameters and carotid intima-media thickness in diabetic patients with the metabolic syndrome: an 18-month prospective study

**DOI:** 10.1186/s12933-016-0480-8

**Published:** 2016-12-03

**Authors:** Manfredi Rizzo, Ali A. Rizvi, Angelo Maria Patti, Dragana Nikolic, Rosaria Vincenza Giglio, Giuseppa Castellino, Giovanni Li Volti, Massimiliano Caprio, Giuseppe Montalto, Vincenzo Provenzano, Stefano Genovese, Antonio Ceriello

**Affiliations:** 1 Biomedical Dept of Internal Medicine and Medical Specialties, University of Palermo, Palermo, Italy; 2 Division of Endocrinology, Diabetes and Metabolism, University of South Carolina School of Medicine, Columbia, SC USA; 3 Department of Biomedical and Biotechnological Sciences, University of Catania, Catania, Italy; 4 Laboratory of Cardiovascular Endocrinology, IRCCS San Raffaele Pisana, Rome, Italy; 5 Department of Human Sciences and Promotion of the Quality of Life, San Raffaele Roma Open University, Rome, Italy; 6 Institute of Biomedicine and Molecular Immunology “Alberto Monroy”, National Research Council (CNR), Palermo, Italy; 7 Department of Internal Medicine, Regional Center for Diabetology, Partinico Hospital, Partinico, Italy; 8 IRCCS MultiMedica, Milan, Italy; 9 Diabetes and Endocrinology, Insititut d’Investigacions Biomèdiques August Pi i Sunyer and Centro de Investigación Biomédica en Red de Diabetes y Enfermedades Metabólicas Asociadas, Hospital Clínic Barcelona, Barcelona, Spain

**Keywords:** Liraglutide, Cardiovascular risk, Carotid intima-media thickness, Metabolic syndrome

## Abstract

**Background:**

Liraglutide, a GLP-1 analogue, exerts several beneficial non-glycemic effects in patients with type-2 diabetes (T2DM), such as those on body weight, blood pressure, plasma lipids and inflammation markers. However, the effects of liraglutide on cardiovascular (CV) risk markers in subjects with the metabolic syndrome (MetS) are still largely unknown. We herein explored its effects on various cardio-metabolic risk markers of the MetS in subjects with T2DM.

**Methods:**

We performed an 18-month prospective, real-world study. All subjects had T2DM and the MetS based on the AHA/NHLBI criteria. Subjects with a history of a major CV event were excluded. One hundred-twenty-one subjects (71 men and 50 women; mean age: 62 ± 9 years) with T2DM and the MetS, who were naïve to incretin-based therapies and treated with metformin only, were included. Liraglutide (1.2 mg/day) was added to metformin (1500–3000 mg/day) for the entire study. Fasting plasma samples for metabolic parameters were collected and carotid-intima media thickness (cIMT) was assessed by B-mode real-time ultrasound at baseline and every 6 months thereafter.

**Results:**

There was a significant reduction in waist circumference, body mass index, fasting glycemia, HbA1c, total- and LDL-cholesterol, triglycerides, and cIMT during the 18-month follow-up. Correlation analysis showed a significant association between changes in cIMT and triglycerides (r = 0.362; p < 0.0001). The MetS prevalence significantly reduced during the study, and the 26% of subjects no longer fulfilled the criteria for the MetS after 18 months.

**Conclusions:**

Liraglutide improves cardio-metabolic risk factors in subjects with the MetS in a real-world study.

*Trial Registration* ClinicalTrials.gov: NCT01715428.

## Background

Liraglutide, a glucagon-like peptide-1 (GLP-1) analogue, is widely used for the treatment of type 2 diabetes mellitus (T2DM). It exerts several glycemic and non-glycemic effects, including the regulation of glucose levels by stimulating glucose-dependent insulin secretion and the suppression of glucagon secretion [1]. Liraglutide is also favorable for body weight by delaying gastric emptying and promoting satiety [2]. In addition, liraglutide has beneficial effects on waist circumference and numerous metabolic parameters including inflammatory markers, blood pressure (BP) and plasma lipids [3], thus improving the cardio-metabolic profile of patients with T2DM [4, 5]. Similar beneficial effects of liraglutide on metabolic parameters were also seen in obese individuals with prediabetes who were at high risk for progression to T2DM and the development of cardiovascular disease (CVD) [6], as well as in subjects who had hypothalamic pituitary injury followed by hyperphagia, in whom several features of the metabolic syndrome (MetS) were present [7].

Liraglutide decreases the prevalence of both MetS and prediabetes in obese, nondiabetic individuals [2, 8], suggesting that this antidiabetic drug might be beneficial in patients with the MetS. It was observed that patients with the MetS responded better to exenatide therapy compared to those without the MetS [9]. Although several clinical trials (reviewed in [3]) suggest that liraglutide, and GLP-1 analogues in general, can beneficially modulate the distinctive features of the MetS, studies performed in patients with diagnosed MetS are lacking. Therefore, we performed an 18-month prospective study in order to assess the effect of liraglutide on cardio-metabolic parameters in subjects with the MetS, collecting real-world data.

## Methods

### Patients and methods

We recruited 121 consecutive subjects (71 men and 50 women, mean age 62 ± 9 years) with the MetS, as diagnosed by the American Heart Association and the National Heart, Lung and Blood Institute (AHA/NHLBI) criteria [10], who were referred for a clinical evaluation to the Unit of Diabetes and Cardiovascular Prevention, University Hospital of Palermo. All subjects had T2DM, were naïve to incretin-based therapies, treated with metformin alone (doses ranging from 1500 to 3000 mg daily), and had HbA1c level >8%. T2DM was a necessary inclusion criteria, since liraglutide is approved in Italy for T2DM therapy only. Therefore, patients included in the present study had, in addition to T2DM, at least 2 of the following criteria [10]: increased waist circumference, increased blood pressure, reduced plasma high-density lipoprotein cholesterol (HDL-C) levels, and increased plasma triglyceride (TG) concentrations (or use of pharmacological therapies for these conditions). Liraglutide was added to metformin, at a dosage of 0.6 mg/day for 2 weeks, followed by a dose of 1.2 mg/day for the rest of the study.

The study design included a medical examination, biochemical analyses and the eco-color-doppler examination of the carotid arteries. The procedures adopted were in agreement with the Helsinki Declaration of 1975 as revised in 1983, and were approved by the Ethics Committee of the University Hospital of Palermo. This study has been registered in clinicaltrials.gov (NCT01715428). Exclusion criteria included the clinical evidence of severe liver dysfunction or renal failure, heart failure, previous major CV events within 12 weeks prior to screening, cancer, and acute infections such as human immunodeficiency virus (HIV), hepatitis B virus (HBV) or hepatitis C virus (HCV). Informed written consent to participate was obtained from all patients before being enrolled in the study. Patients eligible for the study were seen every 6 months for a total period of 18 months. Waist circumference, body height and weight were recorded, and BMI was calculated in kg/m^2^. Obesity was diagnosed when BMI was equal to or greater than 30 kg/m^2^. The primary endpoint of the present study was to investigate the effect of liraglutide on cardio-metabolic parameters. Secondary endpoints included the investigation of subclinical atherosclerosis, as assessed by cIMT, as well as the prevalence of the MetS during the 18-month follow-up.

None of the subjects discontinued metformin or liraglutide, and the dose of liraglutide did not need to be reduced during the study since no patients experienced intolerable adverse effects. Nineteen patients developed mild, transient gastrointestinal symptoms but were able to continue liraglutide throughout the study. Fifteen subjects reported symptomatic hypoglycemia; however, no severe hypoglycemic episodes (based on the joint American Diabetes Association and the Endocrine Society definition) were recorded [11]. The dosages of antihypertensive medications, lipid- lowering agents, and aspirin remained largely unchanged during the course of the study. Data regarding 121 consecutively recruited patients was included in the study. Four patients who discontinued liraglutide therapy within the first 2 weeks of administration were excluded from the analysis. The high rate of adherence to treatment was maintained by bimonthly phone calls to all patients during the entire study.

### Biochemical analyses

All samples were taken after a 14-h overnight fast, and then centrifuged within 30 min of collection, in order to make aliquots of both serum and plasma. All biochemical analyses were made on unfrozen aliquots. All samples were analyzed blinded and simultaneously at baseline and after 6, 12 and 18 months of entry into the study. Serum glucose, glycated hemoglobin (HbA1c), total cholesterol (TC), TG, and HDL-C were measured by routine laboratory methods, while low-density lipoprotein cholesterol (LDL-C) was calculated using the Friedewald formula.

### Color doppler ultrasound of carotid arteries

B-mode real-time ultrasound was performed at baseline and after 6, 12 and 18 months to evaluate the carotid artery wall thickness. All the examinations were performed by a single examiner (A.M.P.) in a blinded manner using the SonoAce Pico Ultasound System (Samsung Medison Co., Korea). The examiner did not have access to previous scans when follow-up studies were performed. The ultrasound examination was performed in a standardized manner with fixed angles of insonation.

As previously reported [5], patients were examined in the supine position, and each carotid wall or segment was examined to identify the thickest intimal-medial site. Each scan of the common carotid artery began just above the clavicle, and the transducer was moved to the carotid bifurcation and along the internal carotid artery. Three segments were identified and measured in anterior and posterior planes on each side: the distal 1.0 cm of the common carotid artery proximal to the bifurcation, the bifurcation itself, and the proximal 1.0 cm of the internal carotid artery. At each of these sites we determined the cIMT, defined as the distance between the echogenic line representing the intimal blood interface and the outer echogenic line representing the adventitial junction. The maximum cIMT value was used for analysis and determined as the mean of the maximum cIMT of near- and far-wall measurements of both the left and right side arteries for each of the 3 arterial segments. We calculated the coefficient of variation for repeated scans to be below 5.0% for all scans, consistent with that found in previous studies [12].

## Statistical analysis

Statistical analysis was performed using SPSS software (V.17.0 for Windows, SPSS Inc., Chicago, USA). All the investigated parameters were assessed for normal vs non-normal distribution by the Kolmogorov–Smirnov normality test. Univariate analysis was performed using paired *t* test to estimate the difference between baseline and the final measurement at each 6 months. Chi squared test was used to assess if there was any association between cIMT changes and the disappearance of the MetS at the end of the study. ANOVA was used to evaluate changes in cardio-metabolic parameters from baseline to 6, 12, and 18 months of liraglutide treatment (p for trend), while correlation analysis was performed using the Spearman rank correlation method. Multivariate analysis (by multiple regression model) was performed in order to determine the independent effect of clinical and laboratory parameters on changes in cIMT.

## Results

**Fig. 1:**
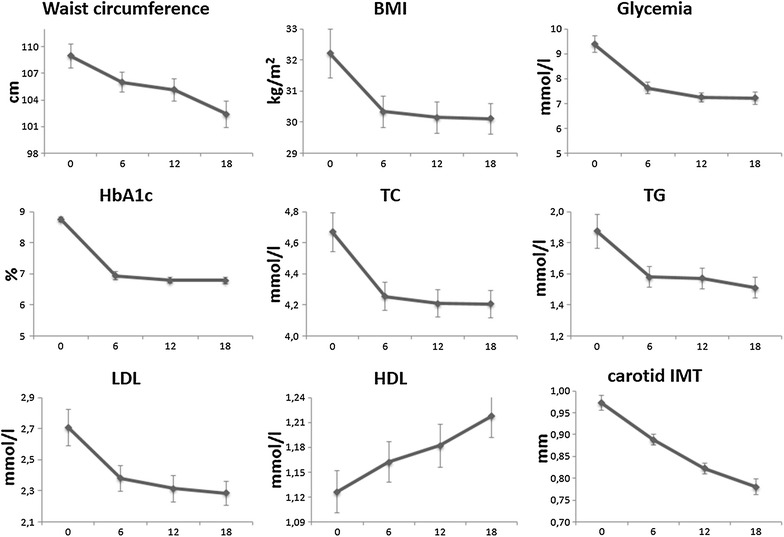
Changes in cardio-metabolic parameters during the study. *BMI* body mass index, *HbA1c* glycated hemoglobin, *TC* total cholesterol, *TG* triglyceride, *LDL* low-density lipoprotein, *HDL* high-density lipoprotein, *IMT* intima media thickness. The data are presented as mean ± SE

Baseline characteristics of the patients are shown in Table 1. The effects of adding liraglutide on all evaluated parameters are shown in Table 2. We found a significant reduction in waist circumference (p = 0.0052), BMI (p = 0.0303), fasting glycemia (p < 0.0001) and HbA1c (p < 0.0001). Regarding plasma lipids, liraglutide therapy significantly decreased TC, TG and LDL-C (p = 0.0017, p = 0.0061 and p = 0.0039, respectively), while HDL-C increased, although the difference did not reach the statistical significance (p = 0.0853). Carotid IMT also reduced over the time (p < 0.0001). The trends in these metabolic parameters over the course of the study are shown in Fig. 1.

**Table 1:** Patients’ baseline characteristics (n = 121)

Age (years)	62 ± 9
Women, n (%)	50 (41)
Diabetes duration (years)	9 ± 8
Smoking habit, n (%)	30 (25)
Past history of cardiovascular diseases n (%)	17 (14)
Past history of cerebro-cardiovascular diseases n (%)	10 (8)
Family history of cardiovascular diseases, n (%)	74 (61)
Systolic blood pressure (mmHg)	128 ± 11
Diastolic blood pressure (mmHg)	79 ± 8
Hypertension, n (%)	93 (77)
Obesity, n (%)	68 (56)
Dyslipidemia, n (%)	93 (77)
Use of anti-hypertensive therapies	
Beta-blockers, n (%)	18 (15)
Angiotensin-converting enzyme inhibitors, n (%)	29 (24)
Calcium entry blockers, n (%)	12 (10)
Diuretics, n (%)	28 (23)
Use of lipid-lowering drugs	
Statins, n (%)	57 (47)
Fibrates, n (%)	3 (3)
Omega-3 fatty acids, n (%)	14 (13)
Aspirin use, n (%)	43 (35)

*n* number

**Table 2:** Effect of liraglutide on cardio-metabolic parameters in all patients (n = 121)

	Baseline	6 months	12 months	18 months	p
Body weight (kg)	86 ± 19	83 ± 18^c^	82 ± 18^c^	82 ± 17^c^	0.2142
CV (cm)	109 ± 15	106 ± 12^c^	105 ± 14^c^	102 ± 16^c^	*0.0052*
BMI (kg/m^2^)	32 ± 9	30 ± 5^b^	30 ± 5^b^	30 ± 5^b^	*0.0303*
Glycemia (mmol/l)	9.38 ± 3.55	7.62 ± 2.50^c^	7.25 ± 2.08^c^	7.21 ± 2.66^c^	<*0.0001*
HbA1c (%)	8.76 ± 0.93	6.94 ± 1.38^c^	6.79 ± 1.02^c^	6.79 ± 1.13^c^	<*0.0001*
TC (mmol/l)	4.67 ± 1.37	4.25 ± 1.00^b^	4.21 ± 0.97^c^	4.21 ± 0.96^c^	*0.0017*
TG (mmol/l)	1.87 ± 1.20	1.58 ± 0.73^b^	1.57 ± 0.75^b^	1.51 ± 0.72^c^	*0.0061*
LDL-C (mmol/l)	2.71 ± 1.30	2.38 ± 0.90^b^	2.31 ± 0.92^c^	2.29 ± 0.84^c^	*0.0039*
HDL-C (mmol/l)	1.13 ± 0.28	1.16 ± 0.27	1.18 ± 0.29^a^	1.22 ± 0.29^b^	0.0853
cIMT (mm)	0.97 ± 0.18	0.89 ± 0.14^c^	0.82 ± 0.13^c^	0.78 ± 0.20^c^	<*0.0001*

Data are presented as mean ± SD

*CV* waist circumference, *BMI* body mass index, *HbA1c* glycated hemoglobin, *TC* total cholesterol, *TG* triglyceride, *LDL* low-density lipoprotein, *HDL* high-density lipoprotein, *cIMT* carotid-intima media thickness

Statistically significant p values are in italics

^a^p < 0.05

^b^p < 0.01

^c^p < 0.001 (all vs baseline)

**Fig. 2:**
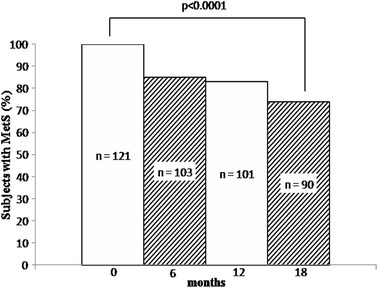
Prevalence of the MetS among all patients (n = 121) during the study. *MetS* metabolic syndrome, *n* number

We performed Spearman correlation analysis in order to search for potential associations between changes in all the evaluated metabolic parameters after 18 months of liraglutide treatment (data not shown). We found that the reduction in HbA1c was significantly correlated with changes in body weight (r = 0.203; p = 0.025), waist circumference (r = 0.272; p = 0.003), BMI (r = 0.187; p = 0.040), fasting glycemia (r = 0.407; p < 0.0001), HDL-C (r = −0.225; p = 0.013) and TG (r = 0.192; p = 0.035). We also found that the reduction in glycemia was significantly associated with changes in TC (r = 0.233; p = 0.010), TG (r = 0.207; p = 0.022) and LDL-C (r = 0.242; p = 0.008). Changes in plasma lipids significantly correlated to each other, while the reduction in cIMT was significantly correlated with changes in TG (r = 0.362; p < 0.0001). Finally, the prevalence of the MetS significantly reduced during the study (p < 0.0001, Fig. 2) and the 26% of subjects no longer fulfilled the criteria for the MetS after 18 months of liraglutide treatment.

We also performed multivariate analysis (data not shown) in order to assess the independent effect of clinical and laboratory parameters on changes in cIMT, and we found that only TG had a predictive role (p = 0.0002).

## Discussion

There is a close association between T2DM and the incidence or development of the MetS. The presence of abnormal metabolic parameters such as central obesity, dyslipidemia, and hypertension further increase the risk of CVD in both T2DM and MetS [13], which remains elevated despite intensive pharmacological treatment [14]. In this 18-month prospective, real-world study we found that liraglutide improved several cardio-metabolic risk factors, including cIMT, in patients with T2DM and the MetS.

Several studies have shown that liraglutide has a positive effect on anthropometric parameters, in particular on body weight (as seen in the LEAD [Liraglutide Effect and Action in Diabetes] studies (reviewed in [3]) and waist circumference [15]. Liraglutide’s benefit was noted even in non-diabetic subjects [2, 8] and in those at high CV risk [16]. In the present study liraglutide progressively reduced waist circumference and BMI over time. Body weight significantly reduced after 6 months of therapy; however, no further change was found during the subsequent 18-month follow-up. A greater liraglutide effect on waist circumference, rather than BMI, may be linked to a potential more powerful effect of liraglutide on abdominal obesity. In this context, there is evidence that the addition of liraglutide to insulin is able to reduce waist circumference more than just increasing the insulin dosage in obese T2DM patients [17].

Moreover, several studies in the last few years have demonstrated a beneficial effect of liraglutide on body weight, independently of the effect on glucose metabolism; such studies typically used greater daily doses of liraglutide than those approved for the management of T2DM. Astrup et al. [2] reported a significant reduction in body weight after 20 weeks of liraglutide therapy in obese non-diabetic subjects with a dose of 3.0 mg/day—a higher amount than what was used in the present study (1.2 mg/day). However, in Japanese patients with T2DM, the reduction in body weight induced by liraglutide was maintained up to 2 years with much lower doses of the drug (ranging from 0.3 to 0.9 mg/day) [18].

In the present study we found a strong and significant reduction in glucose levels as well as a significant reduction in plasma lipids, consistent with established data [8, 19–21]. These latter findings are supported by the positive correlation between improvements in fasting glycemia and plasma lipids (namely TC, TG and LDL-C) as well as by the significant correlation between changes in HbA1c and TG. There was also an 8% increase in HDL-C, although it did not achieve statistical significance. The LEAD studies similarly showed a non-significant increase in HDL-C with liraglutide administration [21, 22]. More recently, the multicenter ROOTS study, which followed 245 diabetic subjects on liraglutide therapy (1.2 or 1.8 mg) for 12 months, showed a significant reduction in TC and LDL-C without changes in HDL-C [23].

We also evaluated the effect of liraglutide on cIMT as a surrogate marker of subclinical atherosclerosis. Some studies have shown that assessing the number of markers of MetS is more useful than the binary definition of MetS for predicting the atherosclerotic burden [24]. We found in the present study a significant association between cIMT changes and the disappearance of the MetS, highlighting the role of subclinical atherosclerosis in such high-risk patients (data not shown). Evidence from pre-clinical studies suggests that liraglutide may have beneficial effects on atherosclerotic plaque formation, progression and its stability [25], in addition to cardioprotective effects leading to improved outcomes and enhanced survival after acute myocardial infarction [26]. In the present study liraglutide significantly reduced cIMT in patients with the MetS during the 18-month follow-up, with the reduction gaining statistical significance after only 6 months of treatment.

The rapid regression of cIMT with the use of liraglutide, previously reported by our group [5] has generated great interest. Liraglutide has been shown to reduce cIMT in a stronger fashion than other incretin-based therapies, including sitagliptin, vildagliptin and exenatide (as reported in the meta-analysis by Song et al. [27]). The macrovascular benefits of liraglutide have been demonstrated with the publication of the Liraglutide Effect and Action in Diabetes: Evaluation of Cardiovascular Outcome Results (LEADER) study [28]. LEADER investigators have suggested that the regression in atherosclerosis induced by liraglutide may have contributed to the significant CV benefit. In the present study, liraglutide therapy was associated with normalization of cIMT values into a normal range; note that the joint European Society of Hypertension/European Society of Cardiology guidelines [29] consider cIMT <0.9 mm as normal. Since cIMT is a surrogate marker of early, subclinical atherosclerosis [30], our findings showing regression of cIMT with the use of liraglutide in MetS patients seem to be congruent with the emerging CV actions of liraglutide.

Interestingly, we also found a significant association between changes in cIMT and those in TG and, at multivariate analysis, TG was the only parameter with a predictive role for changes in cIMT. It is known that TG-metabolism plays a central role in CVD risk [31] and higher TG levels in T2DM subjects are associated with less favorable cardio-metabolic phenotypes and subclinical atherosclerosis [32], that may lead to increased CVD mortality [33]. Additionally, antioxidative markers may be inversely associated with TG levels in early preclinical atherosclerosis even with normal levels of plasma cholesterol [34], and our group has previously demonstrated that liraglutide is able to reduce markers of oxidative stress in patients with T2DM [35]. Kim SK et al. [36] have interestingly reported that nonalcoholic fatty liver disease (NAFLD) is very common in subjects with T2DM, but NAFLD not accompanied by insulin resistance is not associated with a carotid atherosclerotic burden. However, in this latter study [36], coexistence of NAFLD and insulin resistance seemed to be an independent predictor of increased cIMT. Therefore, a greater cIMT reduction in subjects with NAFLD and insulin resistance in our study remains a strong possibility.

As shown in Fig. 2, the prevalence of the MetS significantly decreased during the study. This finding is of considerable clinical value, and we observed a higher decrease in the prevalence of the MetS in comparison with the results reported by Astrup et al. [8], where a non-significant decrease of 12% was recorded. However, it should be highlighted that in the latter study all subjects were obese and only 21 (4%) had diabetes, while in our study 56% of subjects were obese and all of them had diabetes. In this context, a recent study showed that patients with the MetS were more likely to respond in a favorable manner to GLP-1 receptor agonist therapy (in that case, exenatide) than to a dipeptidyl peptidase 4 inhibitor [9].

Since the effect of liraglutide on cardio-metabolic parameters in patients with the MetS has not been evaluated, our data may have important clinical implications. The significant cardio-metabolic effect that we report with the use of liraglutide in the MetS is even higher than what was reported in most of the published studies with the use of this drug in T2DM patients [37]. This finding is of considerable interest, and it is consistent with that reported by Fadini et al. with the use of another GLP-1 analogue in an Italian cohort of patients. In the latter study, MetS patients had a better response to exenatide treatment than those without the MetS [9]. We hope that our findings will help pave the way for future research in the field.

Although liraglutide has been extensively studied in observational studies and clinical trials in cohorts of T2DM patients, to our knowledge ours is the first study directly investigating the effect of liraglutide on several cardio-metabolic risk factors in MetS subjects. It is likely that many subjects included in previous studies involving liraglutide fulfilled the criteria for the MetS; however, the uniqueness of the present study lies in the reporting of novel data that contribute to the understanding of the cardio-metabolic effects of liraglutide beyond glycemic control. Strengths of our study include the real-world setting, 18-month follow-up period, blinded measurements of cIMT, and high compliance rate with liraglutide therapy. Although the use of concomitant medications and CV agents was extensive, their doses were kept largely unchanged during the course of the study in order to minimize potential confounding effects. Further, multivariate analysis found no potential impact of concomitant therapy on changes in cIMT.

A limitation of our study stems from the lack of a control group with a metformin-only arm. However, previous studies have shown that metformin therapy has little or no impact on cIMT [38, 39], and at best, only a modest effect on waist circumference, weight or BMI [40, 41], and lipids [42, 43]. It is therefore unlikely that metformin was responsible for the significant modulation in anthropometric variables, plasma lipids and cIMT that we found in our study. A potential interpretation of the findings is that liraglutide’s effect on cIMT might have been mediated by significantly improved glycemic and metabolic profile; however, no significant correlations between changes in cIMT and those in metabolic parameters were evident. We cannot therefore exclude that the significant reduction in cIMT may be linked to favourable liraglutide effects beyond glycemic control, such as those on oxidative stress, inflammation and atherogenic lipoproteins, although not measured in the present study [3]. Finally, the statistical analysis revealed that the effect of liraglutide on several parameters, including cIMT, was very strong and highly significant; therefore, the probability of the occurrence of our findings by chance alone is extremely low.

## Conclusion

This is the first report of a study prospectively investigating the effects of liraglutide on cardio-metabolic risk factors in subjects with the MetS. Liraglutide improved waist circumference, glucose parameters, plasma lipids and cIMT in an 18-month follow-up, although a major limitation is the lack of a control group. Our findings are of considerable clinical value, but their direct impact on CV outcome remains to be elucidated. The recently published liraglutide CV outcome study reported that liraglutide was able to reduce death from CV causes, nonfatal myocardial infarction and nonfatal stroke in patients with T2DM [28]. Whether this significant CV protection by liraglutide can be extended in patients with T2DM and the MetS, as those included in our study, is at present unknown.

## Abbreviations

AHA/NHLBI: American Heart Association and the National Heart, Lung and Blood Institute
BMI: body mass index
BP: blood pressure
cIMT: carotid-intima media thickness
CVD: cardiovascular disease
GLP-1: glucagon-like peptide-1
HbA1c: glycated hemoglobin
HBV: hepatitis B virus
HCV: hepatitis C virus
HDL-C: high-density lipoprotein cholesterol
HIV: human immunodeficiency virus
LDL-C: low-density lipoprotein cholesterol
LEAD: Liraglutide Effect and Action in Diabetes
LEADER: Liraglutide Effect and Action in Diabetes: Evaluation of Cardiovascular Outcome Results
MetS: metabolic syndrome
NAFLD: nonalcoholic fatty liver disease
T2DM: type 2 diabetes mellitus
TC: total cholesterol
TG: triglyceride
